# Reactivity of Isolated Arteries After 5-Week-Lasting Period of Intermittent Fasting Followed by the Return to Ad Libitum Regimen in Healthy Rats Fed With Normal and High-Fat Diet

**DOI:** 10.33549/physiolres.935719

**Published:** 2025-12-01

**Authors:** Anna ZEMANČÍKOVÁ, Jozef TÖRÖK, Miroslava KVANDOVÁ, Maximilián OLOS, Peter BALIŠ

**Affiliations:** 1 Institute of Normal and Pathological Physiology, Centre of Experimental Medicine, Slovak Academy of Sciences, Bratislava, Slovakia; 2 Faculty of Physics, Ludwig Maximilian University of Munich, Germany

**Keywords:** Intermittent fasting, Rat arteries, Perivascular adipose tissue, Endothelium-dependent relaxation, Adrenergic contraction

## Abstract

Intermittent fasting (IF) represents one of the dietary regimens being effectively used in non-pharmacological prevention and treatment of cardiometabolic disorders. The aim of the present study was to detect the retained alterations at the level of arterial function caused by a 5-week-lasting period of IF in adult male Wistar-Kyoto rats after their switching back to ordinary feeding (4 weeks of *ad libitum* regimen). The rats were administered a diet containing normal or high percentage of fat. Control rat groups were fed continuously *ad libitum*. The decreased weekly calorie intake in rats during IF period was associated with the discontinuation of body weight gain, irrespective of the type of diet; moreover, rats fed with a high-fat diet had significantly increased systolic blood pressure in comparison with the other groups. At the end of the experiment, large and small arteries were isolated from the rats and arterial rings with intact or removed perivascular adipose tissue (PVAT) were prepared for isometric tension recording. In the rat groups exposed to IF period, the aorta rings with intact PVAT showed a significant increase in relaxation responses when compared to groups without IF. The effect of IF was also manifested in the increase in sensitivity of arterial preparations to noradrenaline which was, however, mostly attenuated by the enhanced anticontractile influence of PVAT. These results indicate that the improvement of PVAT properties could represent one of the mechanisms by which IF-induced beneficial effects on vascular function might be preserved even after the return to *ad libitum* regimen.

## Introduction

Intermittent fasting (IF) represents a scheme of food restriction which includes regular fasting periods of various lengths alternating with time windows intended for food intake. This regimen is often used for targeted reduction of body weight, but it is also a part of maintaining a healthy lifestyle including cardiovascular health. It was revealed that intermittent energy deprivation elicits evolutionarily conserved, adaptive cellular responses that are integrated between and within organs in a manner that improves glucose regulation, increases stress resistance, and suppresses inflammation. It has been reported that 12–24 h of fasting results in depletion of hepatic glycogen, accompanied by a switch to a metabolic mode in which free fatty acids and fat-derived ketone bodies are used as energy sources [1]. These substances, together with the fasting-induced changes in the ratios of bioenergetic sensor levels (NAD^+^ to NADH, ATP to AMP, acetyl CoA to CoA), can activate downstream proteins that regulate cell function and stress resistance: transcription factors (peroxisome proliferator–activated receptor γ coactivator 1α, nuclear factor erythroid 2–related factor 2), kinases (AMP-dependent protein kinase), and deacetylases (sirtuins). Changes in these major cellular pathways have profound effects on systemic metabolism and organ function [2–7], and their pharmacological activation is investigated in the treatment of various metabolic, cardiovascular, neurodegenerative, and other disease states [8–9]. As confirmed by many studies on humans and animal models, the IF regimen decreases blood pressure and improves vascular function, including endothelium-dependent relaxant capacity [10–11]; it might reduce cardiovascular sympathetic overdrive and improve the parameters of heart function [12–14]. However, the permanency of these beneficial effects after switching back to ordinary *ad libitum* eating has not been sufficiently clarified yet.

The aim of this study was to evaluate the retained changes in vascular reactivity induced by a 5-week-lasting IF period in healthy male rats after their switching back to *ad libitum* feeding. The measurements were mainly focused on endothelium-dependent relaxation and on sympathoadrenergic contractile responses in isolated large and small arteries. Additionally, the role of intact perivascular adipose tissue in these reactions was examined. Moreover, the effect of the transient IF regimen was compared between normal- or high-fat diet-fed rats.

## Methods

### Experimental animals

The study was conducted in accordance with the European Guidelines on Laboratory Animal Care, and approved by the State Veterinary and Food Administration of the Slovak Republic (protocol code: Ro 4501-3/2020–220). The experiments were performed on adult 18-week-old male Wistar-Kyoto rats, housed at 22–24 °C under a 12:12-h dark-light cycle (06.00–18.00 h light phase) with free access to drinking water. Rats were administered either a control diet (CTD; 10 % energy from fat, formula C 1090–10, Altromin Spezialfutter, Lage, Germany), or a high-fat diet (HFD; 45 % energy from fat, formula C 1090–45, Altromin Spezialfutter, Lage, Germany) for 10 weeks of the experiment (from 18^th^ to 28^th^ week of life). The rats received the diet (CTD or HFD) either in continuous *ad libitum* regimen, or with the transient period of intermittent fasting in the following manner: 1 week of adaptation to the special diet + 5-week-lasting intermittent (alternate-day) fasting + 4-week-lasting *ad libitum* regimen.

During the experiment, systolic blood pressure and heart rate were measured biweekly in conscious animals using the non-invasive tail-cuff method. At the end of the treatment, rats were sacrificed by decapitation after brief CO_2_ anesthesia in a fasted state. Body weight, relative heart and liver weights, and retroperitoneal fat weight were determined in each rat.

Trunk blood was collected after decapitation into pre-heparinised tubes (20 IU/mL of blood) and subsequently subjected to centrifugation (1200 × g, 10 min, RT; Centrifuge 5430 R, Eppendorf, Hamburg, Germany). Plasma was stored at −80 °C until analysis and subsequently thawed at room temperature prior to use. To determine plasma glucose concentration, 100 μL of plasma was loaded onto reagent discs (General Chemistry IV, Celercare, MNCHIP Technologies, Tianjin, China).

### Reactivity of isolated large (conduit) and small (resistant) arteries

Thoracic and abdominal parts of the aorta, large (superior) and small mesenteric arteries, and femoral artery were isolated from individual rats, collected in cold modified Krebs solution with the following composition (in mmol/l): NaCl 118, KCl 5, CaCl_2_ 2.5, MgSO_4_ 1.2, KH_2_PO_4_ 1.2, NaHCO_3_ 25, glucose 11, CaNa_2_EDTA 0.03, and prepared for isometric tension recording.

Paired arterial rings, one with intact and the other with removed perivascular adipose tissue, were prepared from each artery. The endothelial layer was kept intact in all preparations. The arterial rings were suspended in organ chambers with oxygenated (95 % O_2_ + 5 % CO_2_) modified Krebs solution maintained at 37 °C.

For the measurement of reactivity in large arteries, a force-displacement transducer (FSG-01, MDE GmbH, Budapest, Hungary) connected to a NI USB-6221 AD converter (MDE GmbH, Budapest, Hungary) was used, and data acquisition was facilitated through the S.P.E.L. Advanced Kymograph software (version 3.97; MDE GmbH, Budapest, Hungary). Before the measurement, the preparations were equilibrated under a resting tension of 10 mN for 60 min.

The rings of small arteries were positioned in the chambers of wire myographs (Dual Wire Myograph system 410A and 520A and Multi Myograph System 620M and 630MA, Danish Myo Technology A/S, Aarhus, Denmark), with vascular reactivity monitored *via* LabChart 8 software (AD Instruments NZ Limited, Dunedin, New Zealand).

To evaluate endothelium-dependent arterial relaxation, the cumulative concentration-dependent responses of thoracic aorta rings to acetylcholine were assessed as a percentage of the steady-state noradrenaline (10^−6^ mol/l) precontraction.

Adrenergic contractions were determined in abdominal aortas, superior and small mesenteric arteries, and femoral arteries as the responses to cumulatively applied exogenous noradrenaline, and as the responses to cumulatively applied sympathomimetic drug tyramine. Tyramine induces the release of endogenous noradrenaline from the preserved sympathetic neuronal varicosities in the vessel wall; therefore, the level of tyramine contractile responses reflects mainly the amount of endogenously stored sympathetic neurotransmitter noradrenaline, pointing out the richness of arterial sympathetic innervation [15]. In contrast, responses to exogenous noradrenaline indicate the sensitivity and capacity of arteries to contract in response to noradrenaline. Contractile responses were expressed as the active wall tension in mN and normalized to the length of the respective ring (mm).

Noradrenaline was purchased from Zentiva (Prague, Czech Republic); the other chemicals were purchased from Sigma-Aldrich (St Louis, MI, USA). All drugs were dissolved in distilled water, and their concentration was expressed as final concentration in the incubation chamber.

### Data analysis

The results are presented as mean values ± SEM. The data were evaluated using analysis of variance (ANOVA). When suitable, a one-way ANOVA was applied. If statistical significance was detected, post hoc pairwise testing with Bonferroni correction was conducted. For the comparison of concentration-response curves, a two- or three-way ANOVA was performed, followed by Bonferroni-adjusted post hoc testing of vertical differences. GraphPad Prism 8.0 (GraphPad Software; San Diego, USA) was used for the statistical analyses. The differences between means were considered significant when *p*<0.05.

## Results

### Biometric parameters

**Fig. 1:**
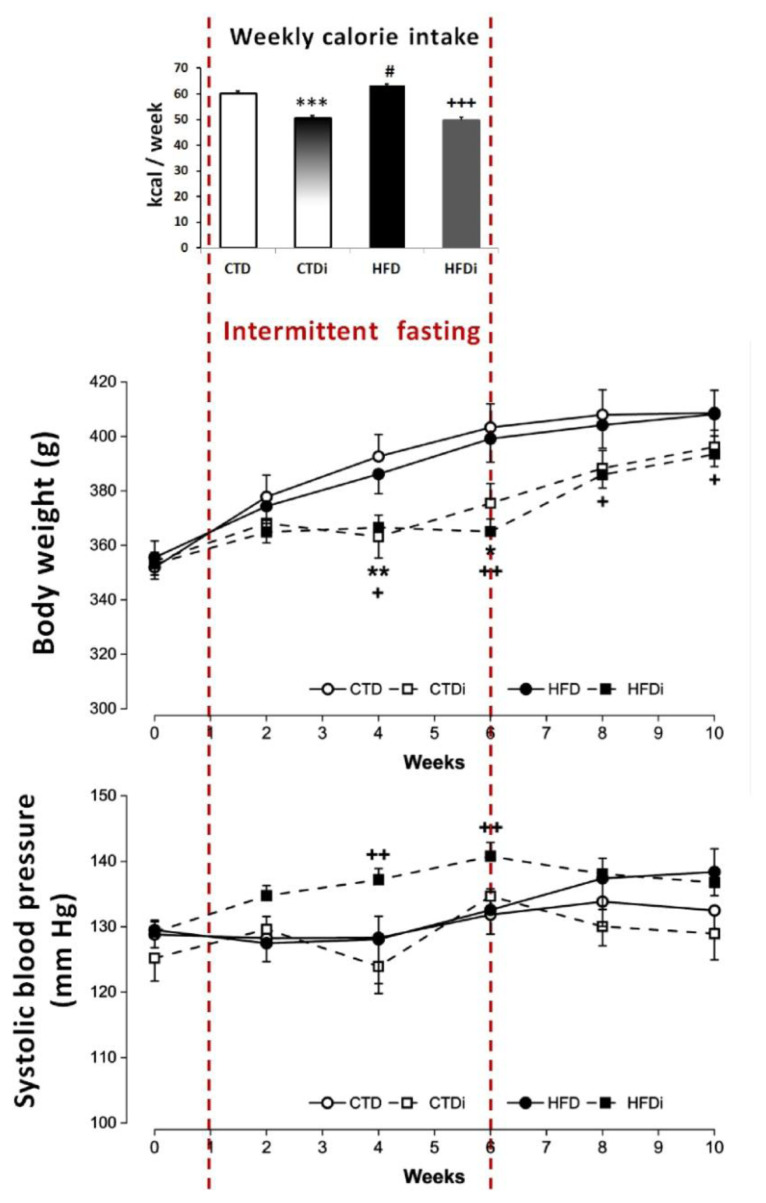
Effect of transient (5-week-lasting) intermittent fasting on weekly calorie intake, body weight and systolic blood pressure in Wistar-Kyoto rats fed with control or high-fat diet. Experimental groups: CTD – control diet administered continuously *ad libitum*; HFD – high-fat diet administered continuously *ad libitum*; CTDi – control diet with transient (5-week-lasting) intermittent fasting; HFDi – high-fat diet with transient (5-week-lasting) intermittent fasting. Symbols represent mean values ± SEM; n = 6–9. *p<0.05, **p<0.01, ***p<0.001 CTDi *vs.* CTD; ^+^*p*<0.05, ^++^*p*<0.01, ^+++^*p*<0.001 HFDi *vs.* HFD; ^#^*p*<0.05 HFD *vs.* CTR.

The high-fat diet (HFD), administered to rats *ad libitum* (AL) for 10 weeks, caused a mild but significant increase in weekly calorie intake (by 5 %) compared to the control diet (CTD). Despite that, it had no significant effect on body weight, retroperitoneal fat mass, plasma glucose, systolic blood pressure, heart rate, or relative heart weight (Fig. 1, Table 1). The groups of rats exposed to 5-week-lasting intermittent fasting (IF) showed a significantly decreased weekly calorie intake during this period – by 16 % in rats fed with CTD and by 21 % in rats fed with HFD – compared to the groups fed with the respective diet in AL regimen. This was associated with the elimination of the increment in body weight, irrespective of the administered diet (Fig. 1). Moreover, during the period of IF, rats fed with HFD had significantly higher blood pressure in comparison with the other experimental groups (Fig. 1). After the return from IF to AL regimen, the lower body weight persisted only in HFD-fed rats (Fig. 1); moreover, at the end of the experiments, both rat groups exposed to transient IF had significantly decreased relative liver weight (Table 1).

### Acetylcholine-induced relaxant responses in isolated thoracic aorta

**Fig. 2:**
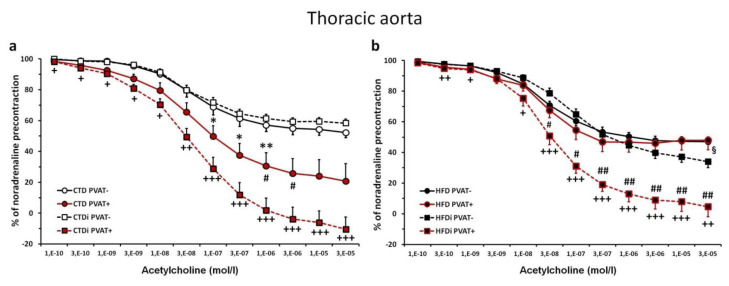
Effect of transient (5-week-lasting) intermittent fasting on acetylcholine-induced relaxant responses in thoracic aortas (with intact (PVAT(+)) or removed (PVAT(−)) perivascular adipose tissue) from Wistar-Kyoto rats fed with control (**a**) or high-fat (**b**) diet. Experimental groups: CTD – control diet administered continuously *ad libitum*; HFD – high-fat diet administered continuously *ad libitum*; CTDi – control diet with transient (5-week-lasting) intermittent fasting; HFDi – high-fat diet with transient (5-week-lasting) intermittent fasting. Symbols represent mean values ± SEM; n = 6–9. *p<0.05, **p<0.01 CTD PVAT(+) *vs.* CTD PVAT(−); ^+^*p*<0.05, ^++^*p*<0.01, ^+++^*p*<0.001 CTDi PVAT(+)/HFDi PVAT(+) *vs.* CTDi PVAT(−)/HFDi PVAT(−); ^#^*p*<0.05, ^##^*p*<0.01 CTD PVAT(+)/HFD PVAT(+) *vs.* CTDi PVAT(+)/HFDi PVAT(+); ^§^*p*<0.05 HFD PVAT(−) *vs.* HFDi PVAT(-).

In continuous AL conditions, the intact perivascular adipose tissue (PVAT) caused significant enhancement of acetylcholine (ACH)-induced endothelium-dependent relaxation in isolated thoracic aortas from rats fed with CTD, but not in aortas from rats fed with HFD (Fig. 2a,b). The 5-week-lasting IF period significantly enhanced the pro-relaxant influence of intact PVAT in thoracic aortas of both, CTD-fed as well as HFD-fed rats, compared to the respective groups with continuous AL regimen (Fig. 2a,b). In rats fed with HFD, the transient IF regimen led to such an improvement of ACH-induced relaxation in PVAT(+) aorta rings that it reached the level of relaxant responses in PVAT(+) rings from CTD-fed rats with the transient period of IF. Moreover, application of IF to HFD-fed rats led to slight but significant increase in maximum relaxant response to ACH in thoracic aorta PVAT(−) preparations, when compared to those from rats fed with HFD continuously AL (Fig. 2b).

### Noradrenaline-induced contractile responses in isolated large (conduit) and small (resistant) arteries

**Fig. 3:**
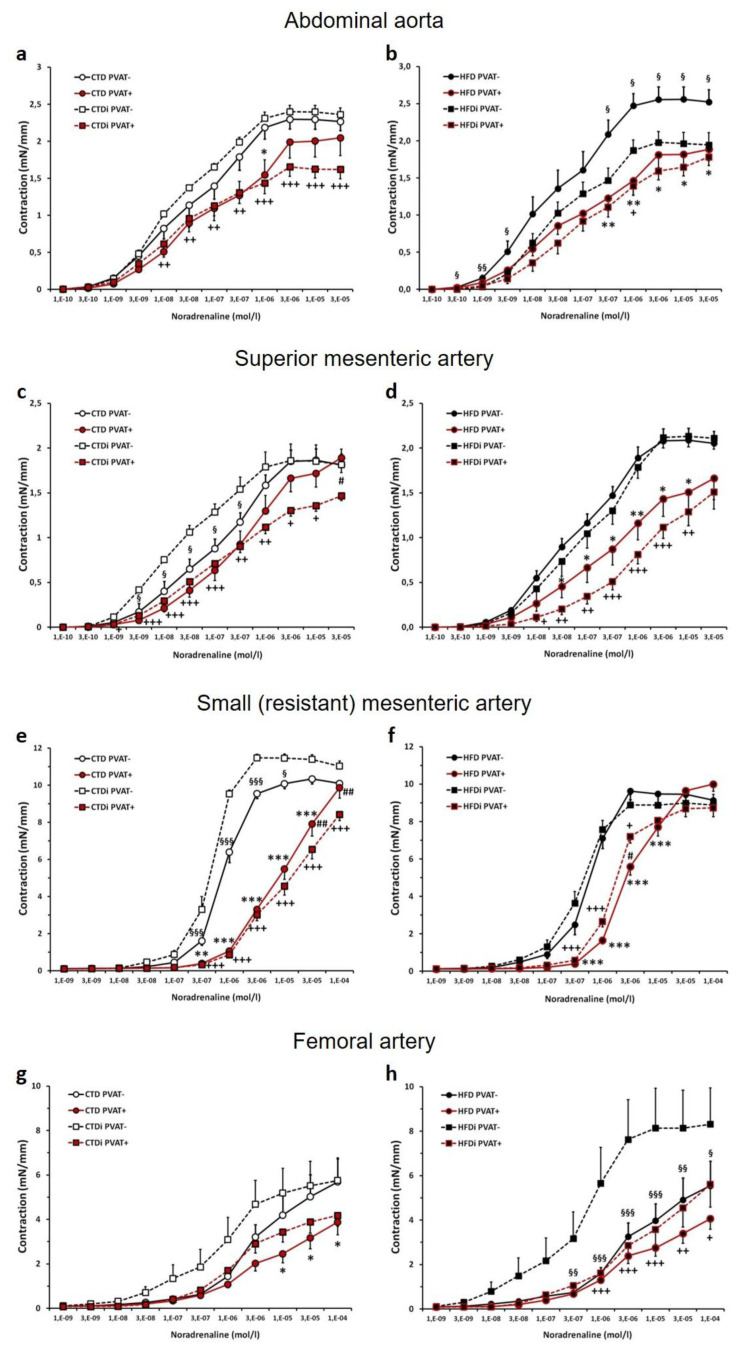
Effect of transient (5﷓week-lasting) intermittent fasting on noradrenaline - induced contractile responses in abdominal aortas (**a, b**), superior mesenteric arteries (**c, d**), small mesenteric arteries (**e, f**) and femoral arteries (**g, h**) (with intact (PVAT(+)) or removed (PVAT(−)) perivascular adipose tissue) from Wistar-Kyoto rats fed with control (a,c,e,g) or high-fat (b,d,f,h) diet. Experimental groups: CTD – control diet administered continuously *ad libitum*; HFD – high-fat diet administered continuously *ad libitum*; CTDi – control diet with transient (5-week-lasting) intermittent fasting; HFDi – high-fat diet with transient (5-week-lasting) intermittent fasting. Symbols represent mean values ± SEM; n = 6–18. *p<0.05, **p<0.01, ***p<0.001 CTD PVAT(+)/HFD PVAT(+) *vs.* CTD PVAT(−)/HFD PVAT(−); ^+^*p*<0.05, ^++^*p*<0.01, ^+++^*p*<0.001 CTDi PVAT(+)/HFDi PVAT(+) *vs.* CTDi PVAT(−)/HFDi PVAT(−); ^#^*p*<0.05, ^##^*p*<0.01 CTD PVAT(+)/HFD PVAT(+) *vs.* CTDi PVAT(+)/HFDi PVAT(+); ^§^*p*<0.05, ^§§^*p*<0.01, ^§§§^*p*<0.001 CTD PVAT(﷓)/HFD PVAT(−) *vs.* CTDi PVAT(−)/HFDi PVAT(−).

The presence of intact PVAT significantly reduced the dose-response contractions to exogenous noradrenaline (NA) in abdominal aortas of all experimental groups (Fig. 3a,b). In PVAT(−) aorta rings of HFD-fed rats exposed to IF period, the dose-dependent NA contractions were significantly decreased compared to PVAT(−) preparations from rats fed with HFD in continuous AL regimen (Fig. 3b).

In superior mesenteric arteries from rats in continuous AL regimen, the attenuating effect of PVAT on NA contractions was detected in HFD-fed rats but not in rats fed with CTD (Fig 3c,d). In CTD-fed group with transient IF regimen, a significant increase in sensitivity to NA was detected in PVAT(−) rings of superior mesenteric artery; however, this was not observed in PVAT(+) rings where the increased anticontractile effect of PVAT caused the dose-dependent contractions to NA to be similar (enhanced in one concentration point) to those in CTD-fed group with continuous AL regimen (Fig. 3c).

In small mesenteric arteries, the intact PVAT had significant inhibitory influence on the dose-response NA contractions irrespective to the type of diet or to the regimen of its administration to rats (Fig 3e,f). The IF period in rats fed with CTD led to significant increase in NA dose-response contractions in PVAT(−) rings of small mesenteric arteries, and to the decrease in these responses in rings with intact PVAT, when comparing to the respective type of rings from rats fed with CTD in continuous AL regimen (Fig 3e). In HFD-fed rats, transient IF only slightly (significantly in one concentration point) increased the sensitivity to exogenous NA in small mesenteric artery PVAT(+) preparations (Fig. 3f).

A trend toward PVAT-mediated inhibition of NA dose-response contractions in femoral arteries was present in all experimental groups, reaching the significance only in the group with CTD administered continuously AL and in the group administered HFD with the transient IF period (Fig 3g,h). The application of IF period produced the tendency to enhanced contractile responses to exogenous NA in rats fed with both types of diet (Fig 3g,h); however, the contractions to NA were significantly higher only in PVAT(−) rings from HFD-fed rats with IF period when compared to PVAT(−) rings from HFD group with continuous AL regimen, and this difference was eliminated by the significant anticontractile effect of PVAT in HFD-treated rats with IF period (Fig 3h).

### Tyramine-induced contractile responses in isolated large (conduit) and small (resistant) arteries

**Fig. 4:**
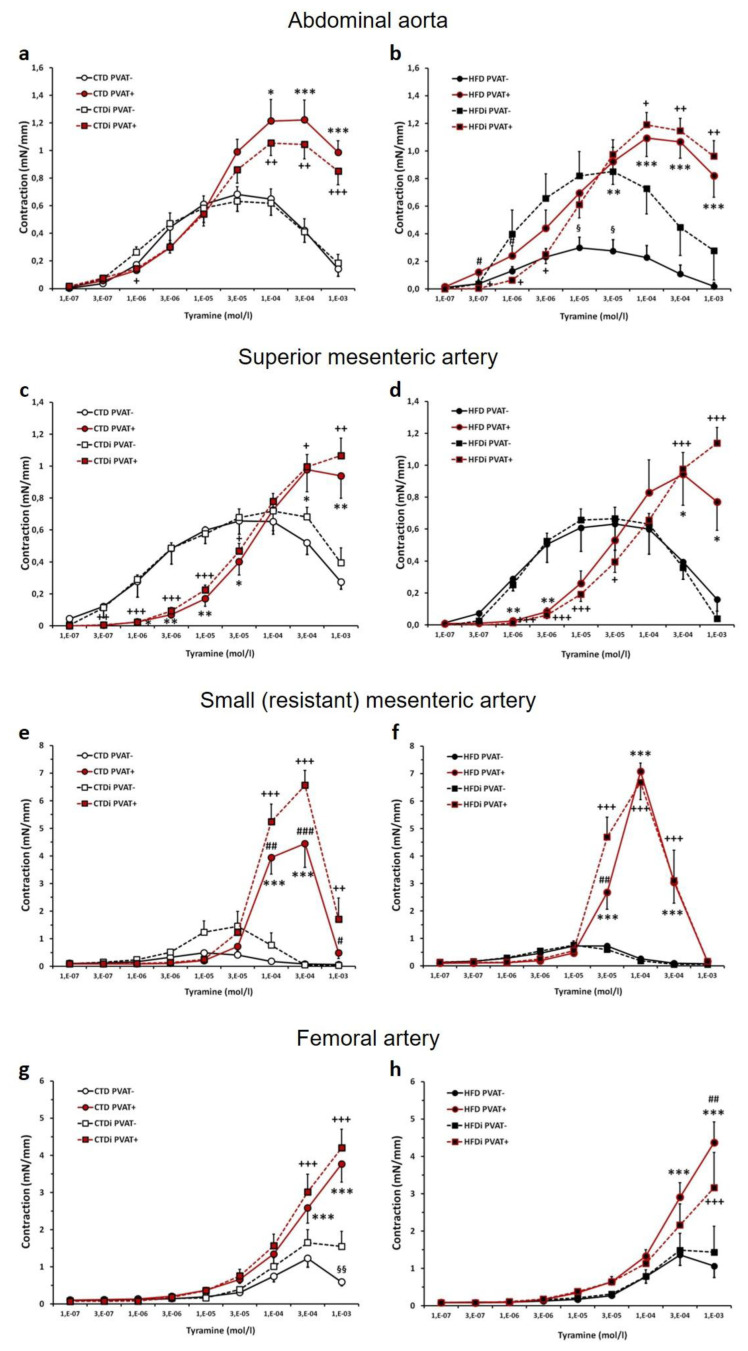
Effect of transient (5-week-lasting) intermittent fasting on tyramine-induced contractile responses in abdominal aortas (**a, b**), superior mesenteric arteries (**c, d**), small mesenteric arteries (**e, f**) and femoral arteries (**g, h**) (with intact (PVAT(+)) or removed (PVAT(−)) perivascular adipose tissue) from Wistar-Kyoto rats fed with control (a,c,e,g) or high-fat (b,d,f,h) diet. Experimental groups: CTD – control diet administered continuously *ad libitum*; HFD – high-fat diet administered continuously *ad libitum*; CTDi – control diet with transient (5-week-lasting) intermittent fasting; HFDi – high-fat diet with transient (5-week-lasting) intermittent fasting. Symbols represent mean values ± SEM; n = 6–18. *p<0.05, **p<0.01, ***p<0.001 CTD PVAT(+)/HFD PVAT(+) *vs.* CTD PVAT(−)/HFD PVAT(−); ^+^*p*<0.05, ^++^*p*<0.01, ^+++^*p*<0.001 CTDi PVAT(+)/HFDi PVAT(+) *vs.* CTDi PVAT(−)/HFDi PVAT(−); ^#^*p*<0.05, ^##^*p*<0.01, ^###^*p*<0.001 CTD PVAT(+)/HFD PVAT(+) *vs.* CTDi PVAT(+)/HFDi PVAT(+); ^§^*p*<0.05, ^§§^*p*<0.01 CTD PVAT(−)/HFD PVAT(−) *vs.* CTDi PVAT(−)/HFDi PVAT(−).

The maximum tyramine (TYR)-induced contractile responses were generally higher in PVAT(+) than in PVAT(−) arterial preparations. This effect was detected also in the abdominal aortas, except for the rat group administered HFD with the transient IF period where the maximum TYR-induced contractions in PVAT(−) rings were significantly increased and approached the level of those with intact PVAT (Fig 4a,b). In contrast, the IF period in rats fed with CTD did not significantly change the maximum responses to TYR in abdominal aorta rings with PVAT both intact and removed (Fig. 4a).

The superior mesenteric arteries with intact PVAT responded to TYR with significantly decreased sensitivity but with the tendency to higher maximal response when compared to PVAT(−) rings. The IF period did not change the course of this reaction in PVAT(−) and PVAT(+) rings of superior mesenteric arteries when compared to the groups with continuous AL regimen with the respective type of diet (Fig. 4c,d).

The contractile responses of small mesenteric arteries to TYR were markedly higher in the presence of PVAT, which was significant in all experimental groups (Fig 4e,f). The TYR-induced contractions of small mesenteric artery PVAT(+) rings were significantly increased due to the transient IF in CTD-fed rats (Fig. 4e). In the group fed with HFD, the IF regimen caused the TYR contractions to be significantly greater only in one concentration point of the dose-response curve in PVAT(+) rings (Fig 4f); however, the significant difference in maximum responses to TYR was not detected between IF- and AL-regimen groups of HFD-treated rats.

The maximum TYR-induced contractions of femoral arteries from all rat groups were significantly higher in PVAT-intact preparations when compared to those with PVAT removed (Fig. 4g,h). The significant increase of TYR-induced contractions in femoral arteries due to transient application of IF was detected only in PVAT(−) rings from CTD-fed rats (Fig. 4g). In contrast, the significant decrease in maximum contraction to TYR in PVAT(+) femoral arteries was observed after the IF period being applied to HFD-fed rats (Fig. 4h).

## Discussion

In the present study, intermittent fasting (IF) in rats during a 5-week-lasting period was associated with a significant reduction in weekly calorie intake and with an elimination of body weight increment. Many previous studies confirmed profound beneficial effect of various regimens of caloric restriction on the cardiovascular system, being manifested in the protection of myocardium from ischemic damage, the prevention of postinfarct cardiac remodelling, the improvement of endothelial function and arterial stiffness, and in the reduction of blood pressure level [10,12,16–18]. In contrast, the present results show no decrease in systolic blood pressure of rats neither during the acute phase of IF, nor after the switching back to *ad libitum* (AL) regimen. In the group of IF-treated rats the endothelium-dependent relaxations of thoracic aorta rings with removed perivascular adipose tissue (PVAT) were not altered in comparison with the rats fed continuously AL. On the other hand, when measured with intact PVAT, the aorta rings from rat groups exposed to IF period show significant increase in relaxation responses when compared to groups without IF. This indicates that IF-induced changes in perivascular fat are long-lasting and could represent one of the mechanisms by which the improvement of vascular function might be preserved even after switching from IF back to AL regimen. Although in IF-treated rats, after their 4-week-lasting return to AL regimen, the persistence of reduced body weight was observed only in the group fed with a high-fat diet (HFD), and retroperitoneal fat weights were not different from those in groups with continuous AL feeding, the IF-produced qualitative changes of adipose tissue might still be retained and influence the function of other organs by altered spectrum of adipocyte-derived substances. It was confirmed in previous studies that fasting and energy restricting diets decrease serum levels of adipocyte-released substances such as leptin or resistin; on the other hand, serum adiponectin was found to be significantly elevated [16,19–20]. Adiponectin is known to exert vasoprotective effects through its direct actions in the vascular system, such as increasing endothelial NO production [21] and reducing the expression of pro-inflammatory cytokines [22]. Therefore, among other mechanisms, the increased release of adiponectin from PVAT might be responsible for the enhanced pro-relaxant effect of PVAT after the period of IF detected in this study. Moreover, irrespective of the type of diet, a significantly reduced relative liver weight was found in this study in rats exhibited to IF, indicating enhanced liver metabolism, as confirmed previously by Ma *et al.* [23] who revealed global elevation of metabolites in the livers of fasting mice. These results suggest that stimulating metabolism in the liver may contribute to the health benefits of fasting and provides one possible approach for non-pharmacological intervention in chronic metabolic diseases, such as non-alcoholic fatty liver disease [24].

Many studies have brought findings that IF has a blood pressure-lowering effect and improves cardiovascular autonomic regulation in healthy humans, as evidenced by the enhanced heart rate variability and the reduced resting heart rate [13,25]. Such observations indicate an IF-produced autonomic shift towards reduced sympathetic drive and, oppositely, to the increase in parasympathetic tone, which might be highly beneficial in patients with hypertension and metabolic syndrome who often manifest a significant autonomic imbalance. To evaluate the possible IF-induced alterations in sympathetic tone at the level of arterial system, the sympatho-adrenergic contractions in isolated large and small arteries of IF-treated rats were measured in this study. The obtained results show inconsistent changes in these responses depending on the arterial type and the type of administered diet; however, in general, it is possible to see a trend towards enhanced sensitivity to exogenous noradrenaline and to increased store of endogenous noradrenaline in arteries (detected as significant increase in tyramine-induced contraction). The possible arterial sympatho-adrenergic overdrive indicated by these results could partially explain the present finding of no decrease in blood pressure or heart rate in rats with IF regimen. Moreover, the question arises whether the exposition to IF could produce inadequate stress to which these animals were not able to adapt, even over a longer period. Although alternate-day fasting is widely used in rats in experimental procedures and majority of results have shown a beneficial effect of this treatment on various aspects of their health and life span, it was documented that the periods of long-lasting food deprivation in rats and other rodents might produce alterations in numerous hormonal and behavioural responses compatible with those seen during stress response reactions [26–27]. This is supported also by the observations of Bucaktepe *et al*. [28] showing increased blood and urine concentrations of catecholamines during a 4-week-lasting experiment simulating Ramadan fasting in rats.

In the present study, the enhanced arterial responses to noradrenaline were, however, mostly attenuated with the increased anticontractile influence of PVAT, as observed in the arterial rings with preserved PVAT, indicating that within the intact organism the IF-induced enhancement of PVAT anticontractile properties may partially compensate the increased sympathetic activity in some arterial beds. This is obviously demonstrated in mesenteric arteries in rat group with control diet exposed to transient IF where the increased pro-relaxant properties of the mesenteric PVAT may serve as a counterbalance to the enhanced adrenergic contractions in order to ensure the increased blood flow and utilization of the food received in the intestine.

One of the aims in the present study was to compare the effect of transient IF regimen on arterial reactivity in rats fed with different diets; *i.e.*, diet with normal or with high percentage of fat. In spite of the mild but significant increase in the weekly calorie intake in HFD-fed rats, the 10-week-lasting administration of HFD *ad libitum* did not produce any significant changes in body weight, systolic blood pressure, and plasma glucose concentration; on the other hand, it caused elimination of the pro-relaxant effect of intact PVAT during acetylcholine-induced relaxation of isolated thoracic aorta, which was observed in the group of rats fed with CTD. The important result of this study is that the transient IF regimen was able to restore the pro-relaxant properties of PVAT in thoracic aortas from HFD-fed rats, which means that in the intact organism the endothelium-dependent relaxation was improved due to IF treatment, even four weeks after the transition to AL regimen. Despite this beneficial IF-caused enhancement of pro-relaxant PVAT influence, which was manifested also in attenuating the exaggerated sympatho-adrenergic contractions in some of the arteries tested in this study, the significant elevation in systolic blood pressure was detected during the period of IF in rats fed with HFD compared to the other groups. This indicates the potential cumulative effect of the negative factors of HFD feeding and the possible adverse aspects of IF treatment in some animal species, such as the stress reaction due to long-lasting food deprivation. However, the mechanisms of this interaction require further investigation.

In conclusion, the results of this study document that the IF regimen in rats exerts beneficial effect on arterial reactivity which persisted even after a 4-week-lasting return to AL regimen, and that this effect is mediated mainly through the enhanced pro-relaxant/anticontractile properties of perivascular fat. This IF-produced effect on PVAT was similar in arteries from CTD- and HFD-fed rats, indicating that this form of treatment might be effectively used in prevention of vascular impairment associated with an unhealthy diet and metabolic syndrome.

## Figures and Tables

**Table 1:** Final values of selected cardiometabolic parameters in the experimental rat groups (at the end of the 10-week-lasting experiment).

	CTD	CTDi	HFD	HFDi
*HR (bpm)*	348.6±9.4	364.7±13.5	339.6±8.2	362.4±18.8
*HW/BW (mg/g)*	3.45±0.06	3.55±0.12	3.33±0.04	3.53±0.08
*LiW/BW (mg/g)*	22.94±0.26	21.65±0.25 **	22.77±0.66	21.43±0.15 **+**
*RFW (mg)*	4348.3±339.6	4588.3±217.7	4072.6±199.7	4284.0±156.8
*Glu (mmol/l)*	7.48±0.62	7.28±0.09	8.66±0.73	7.30±0.13

Abbreviations: HR – heart rate; HW/BW – heart weight to body weight ratio; Li/BW – liver weight to body weight ratio; RFW – retroperitoneal fat weight; Glu – glucose (plasma concentration). Experimental groups: CTD – control diet administered continuously *ad libitum*; HFD – high-fat diet administered continuously *ad libitum*; CTDi – control diet with transient (5-week-lasting) intermittent fasting; HFDi – high-fat diet with transient (5-week-lasting) intermittent fasting. Values represent mean values ± SEM; n = 6–9.

** *p*<0.01 CTDi *vs.* CTD;

+ *p*<0.05 HFDi *vs.* HFD.
